# Impact of role conflicts and self‐efficacy on academic performance of graduate‐entry healthcare students: A lagged study

**DOI:** 10.1111/nhs.12934

**Published:** 2022-03-26

**Authors:** Anne O'Connor, Gemma McCarthy, Deirdre O'Shea

**Affiliations:** ^1^ School of Allied Health University of Limerick Limerick Ireland; ^2^ Health Research Institute University of Limerick Limerick Ireland; ^3^ Kemmy Business School University of Limerick Limerick Ireland

**Keywords:** academic performance, graduate entry, nursing students, role conflict, self‐efficacy, students

## Abstract

Graduate entry healthcare students experience many challenges during their academic journey. The impact of these challenges needs to be considered to support students through their training and education. In this study, we examined the impact of experiencing these role conflicts (at the outset of the academic year), for example, family and caring responsibilities, activities with family/friends, and daily tasks/chores, on the academic performance (at the end of the academic year) of graduate‐entry healthcare students. We also investigated the potential of students' self‐efficacy for learning to mitigate the extent to which such role conflicts impact academic performance. Findings demonstrate that the more graduate entry healthcare students experienced conflicts between their life responsibilities and their academic responsibilities, the worse their academic performance was across the year. This negative relationship was somewhat mitigated by high self‐efficacy for learning. The practical implications of our research suggest the need to provide specific mitigation strategies to support healthcare students regarding conflicts between their life/family responsibilities and their academic work.

**Key points**

- Graduate‐entry students whose life activities conflicted with their academic studies on entry to their program had a lower end‐of‐year academic performance. No such relationship was found for students experiencing conflict in terms of their academic studies interfering with their life activities.
- Students' self‐efficacy for learning buffered the negative effects of the conflict from their life role to their academic work on academic performance. Thus, graduate entry students with higher self‐efficacy for learning in the early stages of their studies were better able to mitigate the impact of such conflict so that their end‐of‐year academic performance was not impacted to the same extent as those with lower self‐efficacy for learning.
- The practical implications of this study suggest the need to provide specific mitigation strategies to support graduate entry healthcare students with regard to conflicts between their life/family responsibilities and their academic work.

## 1 BACKGROUND

Graduate entry and accelerated training programs are becoming popular routes in the training of medical and health professionals, with the advantages and benefits of such graduate entry routes espoused in both research and practice. However, graduate entry students experience many challenges. These students are likely to have significant responsibilities in domains of their lives outside of their studies (e.g., family responsibilities, caring responsibilities, financial responsibilities), and there is potential for conflict between these roles and their academic pursuits. Thus, the impact of these challenges needs to be considered to sufficiently support students through their training and education.

Evidence from medical education suggests that burnout in medical and healthcare professionals may be initiated as early as the academic training stages, with a recent study reporting that 37% of interns (i.e., medical students) transitioning from medical school to the clinical environment met the criteria for psychological distress, reporting high levels of emotional exhaustion, depersonalization, and a low sense of personal accomplishment, which are components of burnout (Hannan et al., 2018). A significant growth in the development of graduate entry health professional programs globally may contribute to these challenges. Many of these programs are accelerated in nature, resulting in heavier workloads to develop the knowledge, skills, and abilities required over a shorter timeframe than traditional undergraduate entry routes. Limited knowledge exists regarding how well graduate entry students transition in the early stages of these academic programs, and whether their academic performance is impacted by challenges such as work–life balance and family commitments. Further research in this area would help to inform health professional education providers and guide graduate entry curricula in healthcare, thus preparing healthcare students for potential challenges through the provision of appropriate supports throughout the academic program.

Many sources of stress exist for third level students at all stages of academia. General stressors include workload and deadlines associated with exams and assessment. Practice placements add further stressors for healthcare students due to the need to balance academic demands and deadlines with work‐based learning needs (O'Connor et al., 2018; Wang et al., 2019). Sleep deprivation, distancing from family and friends, and financial worries have also been identified as stressors (He et al., 2018; Mian et al., 2018). Thus, health professional students frequently juggle different types and contexts of learning, causing significant time pressures and stress that can extend to family and home commitments (Hill et al., 2018; Labrague et al., 2017).

While the benefits of graduate entry level students are well outlined in the literature in terms of the life experience they bring to the health professional field (Gibbons, 2010; Rapport et al., 2009), it is unclear what effect the stressors associated with juggling their studies and their other life activities may have on their academic performance. Developing a better understanding of this would help to steer curriculum content particularly in the early academic years to prepare students to recognize signs of stress and develop skills and strategies to mitigate the negative effects of role conflict. In this research, we draw on the concept of inter‐role conflict where the role pressures from the education and family domains are mutually incompatible in some respect (Greenhaus & Beutell, 1985). For example, role conflicts between work and family contexts have been found to be related to decreased productivity, lost work time, increased health risks for employed parents, poorer performance of the parenting role, absenteeism, poor morale, and depression (Obrenovic et al., 2020; Wang & Tsai, 2014). We investigate whether similar relationships exist when role conflict (between academic activities and other life activities) is experienced during graduate entry health professional training.

Psychological resources of the individual can be important buffers of the stressor–performance relationship. One such personal resource is perceived self‐efficacy, defined as one's belief in one's capabilities to mobilize resources and courses of action to exercise control over events in one's life (Wood & Bandura, 1989). Previous studies suggest there may be potential benefits to focusing on improving student self‐efficacy due to its positive association with factors such as motivation and effective learning strategies which have been directly linked to academic performance (Asikainen & Gijbels, 2017; Prat‐Sala & Redford, 2010). Additionally, self‐efficacy can mitigate the impact of stressors, such as role conflict, on burnout and performance in challenging situations such as healthcare roles, thus highlighting its importance as a personal resource in both undergraduate and postgraduate training of all healthcare workers (Perrewé et al., 2002; Yao et al., 2018). Research has shown that, in different contexts, increased work–life conflict is associated with higher levels of psychological distress and lower levels of psychological well‐being (Bonsaksen et al., 2021; Huat et al., 2018). Given that students who enter graduate programs are more likely to have additional demands in their lives, for example, caring responsibilities, life responsibilities, spouse/partner roles, household chores, and so forth, we investigated the potential impact of this on their academic performance. Drawing on theory and research on work–life conflict, which proposes a bidirectional distinction in the nature of this conflict (Mesmer‐Magnus & Viswesvaran, 2005), we also investigate the extent to which conflict between a student's life responsibilities and demands (e.g., caring and other life responsibilities, etc.) impacts their ability to engage with the requirements of their program (which we term life‐to‐academic program conflict, LAPC) and ultimately on their academic performance. Additionally, we investigate the conflict in the opposite direction also—the time and effort that students must commit to their academic studies may conflict with and take them away from responsibilities and roles in their life more broadly, and so we examine the impact of conflict from one's academic program to one's life in terms of its potential impact on academic performance. We investigate whether self‐efficacy mitigates the potential negative impact of such conflicting roles, by examining whether it moderated the relationships between LAPC and academic program‐to‐life conflict (APLC) on academic performance. Thus, our research hypotheses were as follows:

Hypothesis 1 *Self‐efficacy at the start of the academic year will be positively related to end‐of‐year academic performance*.

Hypothesis 2 *(a) Conflict from one*'*s life to academic studies (LAPC) and (b) from one*'*s academic studies to life (APLC) will be negatively related to end‐of‐year academic performance*.

A high belief in one's ability to cope with the demands of learning what is required in the graduate program (i.e., self‐efficacy for learning) should enable students to cope with any conflicting role demands they may experience. As a result, they will likely experience less anxiety and less effort in ruminating about such conflict, and are more likely to believe they can learn what is required, even in the face of competing demands from different life roles. As such, high levels of self‐efficacy should mitigate the negative effect of role conflicts on students' academic performance.

Hypothesis 3 *Self‐efficacy for learning will moderate the relationship between both types (APLC and LAPC) of conflict at the start of the academic year and end‐of‐year academic performance, such that experiencing either type of conflict will have a weaker negative impact on academic performance when self‐efficacy is high*.

## 2 METHODS

### 2.1 Ethics approval

Approval for this study was granted by the University of Limerick Education and Health Sciences Research Ethics Committee (REF: 2019_09_08_EHS).

### 2.2 Sample and procedure

A lagged study design was employed. All incoming first year students (Academic Years 2019/20 and 2020/21) registered in graduate entry health professional programs in Bachelor of Medicine/Bachelor of Surgery, MSc Occupational Therapy, MSc Physiotherapy, MSc Human Nutrition and Dietetics, or MSc Speech and Language Therapy at an Irish university were eligible to participate, providing a potential sample of 150 students. Each participant was invited to complete a self‐reported questionnaire close to the start of the academic year. In this survey, they were also asked to indicate their consent (or not) to access their academic performance at the end of the same academic year. One hundred and eighteen students took the survey close to the beginning of the academic year in 2019 or academic year 2020 (see Table 1 for descriptive statistics). At the start of the survey process, students were asked to provide permission for the researchers to access their end‐of‐year academic performance, whereupon 66 students agreed. Given that this was our outcome variable, this determined our sample size (*n* = 66) for hypothesis testing and missing data on this variable meant that responses to the initial survey were not considered in the analyses. All responses were anonymized, and student ID was only requested to enable the researchers to match the participants' academic performance to their completed surveys. There was no significant difference between those that gave permission to access their cumulative end‐of‐year grade point average (GPA) and those who did not in terms of APLC, LAPC, or self‐efficacy for learning (*t* = −1.96, *p* = 0.245; *t* = −0.473; *p* = 0.637; *t* = 1.413, *p* = 0.160, respectively).

**TABLE 1:** Descriptive statistics for demographic variables

Variable	M/count	SD/%
Age	25.68	3.96
Gender		
Male	19	17.6%
Female	89	82.4%
Caring responsibilities		
Yes	11	10%
No	99	90%
Relationship status		
Married or in a civil partnership	14	18.2%
Divorced/separated	1	1.3%
Never married	62	80.5%
Highest education level		
Diploma, pass bachelor's degree, or trade qualification	8	7.3%
Honors bachelor's degree	83	75.5%
Master's degree	17	15.5%
PhD or professional doctorate	2	1.8%

To encourage participation, the initial data collection involved a meeting with students where they were informed of the study and the benefits of partaking. An online workshop designed to provide tips and strategies for coping with graduate entry study and workload was also offered to potential participants after they had completed the survey. Two reminders were sent by email after each survey to optimize participation.

### 2.3 Measures

Self‐efficacy was measured at the start of the academic year using the perceived competence for learning questionnaire (Williams & Deci, 1996; Williams et al., 1998), which is a four‐item scale (e.g., “I feel confident in my ability to learn the material”; α = 0.91). Participants responded on a 5‐point Likert scale (1 = strongly disagree; 5 = strongly agree).

Role conflict was assessed at the start of the academic year using the scale developed by Bolino and Turnley (2005), which was adapted so that it reflected the academic rather than the work context. Role conflict comprised two subscales: LAPC (e.g., “The demands of my graduate program interfere with my home and family life”; α = 0.92) and APLC (e.g., “I have to put off doing things in my graduate program because of demands on my time at home”; α = 0.90). Both subscales comprised five items each, and participants were asked the extent to which they would agree with each statement using a 7‐point Likert scale (1 = strongly disagree; 7 = strongly agree).

Academic performance was assessed at the end of the academic year using the cumulative end‐of‐year GPA. This GPA score was subsequently matched to the student's survey responses. The GPA score ranges from 0 to 4.0 with 2.0 indicating a minimum passing GPA. The GPA combines grades across modules, weighted according to the European Credit Transfer credits awarded to a given module.

### 2.4 Data analysis

The statistical package SPSS version 26 was used to conduct all analyses. An add‐on package, PROCESS version 3.5 (Hayes, 2017, Model 1) was used to conduct moderation analyses, which were based on bias‐corrected bootstrapping analysis that yield 95% confidence intervals. To minimize multicollinearity, the predictor variables were mean centered. Testing moderation requires multiplying the independent variable and moderator variable, and which interaction variable by its nature will be correlated with the original predictor variable and moderator. Mean centering subtracts the variable's mean from all observations on that variable so that the variable's new mean is zero, and is recommended when conducting moderation analysis to avoid multicollinearity (Iacobucci et al., 2016). We conducted two regressions using Model 1 of the PROCESS add‐on, both with self‐efficacy as the moderator and end‐of‐year academic performance as the outcome variable. In the first, we modeled LAPC as the predictor and controlled for APLC as a covariate, and in the second, we modeled APLC as the predictor, controlling for LAPC as a covariate. All relevant regression assumptions were met. In the event of a significant moderation effect, we ran simple slopes analyses to illustrate the relationship between role conflict and academic performance when self‐efficacy was low (1 standard deviation below the mean), moderate (mean value of self‐efficacy), and high (1 standard deviation above the mean). There was no significant difference between year groups in terms of academic performance (*t* = −0.221, *p* = 0.826), life to academic programme conflict (LAPC) (*t* = 0.449, *p* = 0.654), Academic Programme to Life Conflict (APLC) (*t* = 1.504, *p* = 0.135), or self‐efficacy (*t* = −0.689, *p* = 492). As such, the data from both academic year cohorts were combined to run the analyses. Moreover, no significant differences in gender were found for any of the variables of interest in this study.

### 2.5 Preliminary analysis

Our analyses are based on the number of participants who provided permission to the researchers to access their end‐of‐year academic performance record (*N* = 66). The Pearson coefficient (*r*) was used to explore initial correlations between academic performance, conflict at each time point, and self‐efficacy. The results were in line with our expectations: Self‐efficacy for learning was positively correlated with academic performance and negatively related with LAPC. Additionally, we found a small negative correlation between academic performance and LAPC (see Table 2). Next, we investigated whether those with caring responsibilities reported higher levels of role conflict at the start of that academic year in comparison to those without caring responsibilities. A one‐way analysis of variance showed that those with caregiver responsibilities reported significantly higher levels of LAPC, *F*(1,106) = 10.00, *p* = 0.002. There was no difference between these groups in terms of APLC.

**TABLE 2:** Pearson correlations between predictor, moderator, and outcome variables

	*M*	SD	*N*	1	2	3
1. End‐of‐year academic performance	3.194	0.330	66	–	–	–
2. Start‐of‐year self‐efficacy	5.318	1.090	118	0.287*	–	–
3. Start‐of‐year life‐to‐academic program conflict (LAPC)	3.209	1.478	117	−0.249*	−0.318**	–
4. Start‐of‐year academic program‐to‐life conflict (APLC)	4.559	1.420	117	−0.057	−0.339**	0.528**

* *p* < 0.05.

** *p* < 0.01.

## 3 RESULTS

### 3.1 Hypothesis testing

Results are presented in Tables 3 and 4. From these, we can see that the findings support Hypothesis 1: Higher self‐efficacy for learning at the start of the year was associated with higher academic performance at the end of the academic year. Conflict from one's life to one's academic studies assessed at the start of the academic year had a negative impact on academic performance at the end of the year, but we found no such relationship for conflict from one's academic program to other life activities. Thus, we found support for Hypothesis 2(a) but not Hypothesis 2(b).

**TABLE 3:** Moderating effect of learning self‐efficacy on the relationship between LAPC and academic performance at the end of the year (*N* = 66)

Variable	*B*	SE B	*t*	*p*
LAPC	−0.080	0.033	−2.46	0.017
Self‐efficacy (SE)	0.082	0.040	2.06	0.044
LAPC × SE	0.057	0.026	2.14	0.037
APLC (covariate)	0.043	0.034	1.26	0.213
Model statistics	*R* ^2^ = 0.203 *F*(4,62) = 3.89; *p* = 0.007			
Test of highest order unconditional interaction	Δ *R* ^2^ = 0.060 *F*(1,61) = 4.57; *p* = 037			

Abbreviations: APLC, academic program‐to‐life conflict at start of year; LAPC, life‐to‐academic program conflict at start of year.

**TABLE 4:** Moderating effect of learning self‐efficacy on the relationship between APLC and academic performance at the end of the year (*N* = 66)

Variable	*B*	SE B	*t*	*p*
APLC	0.042	0.113	1.19	0.238
Self‐efficacy (SE)	0.091	0.035	2.26	0.027
APLC × SE	0.034	0.023	1.44	0.156
LAPC (covariate)	−0.069	0.032	−2.12	0.038
Model statistics	*R* ^2^ = 0.172 *F*(4, 61) = 3.16; *p* = 0.020			
Test of highest order unconditional interaction	Δ *R* ^2^ = 0.028 *F*(1,61) = 2.07; *p* = 0.156			

Abbreviations: APLC, academic program‐to‐life conflict at start of year; LAPC, life‐to‐academic program conflict at start of year.

**FIGURE 1:**
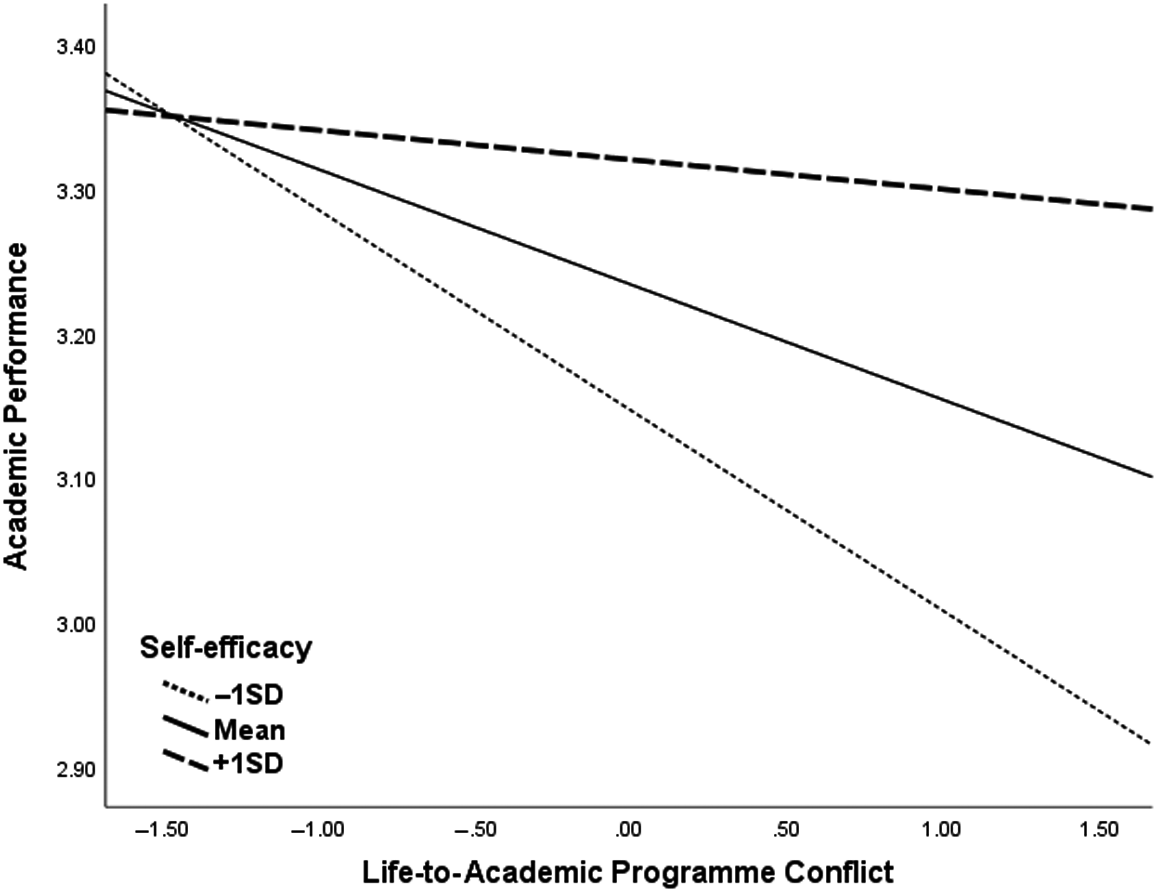
The moderating effects of self‐efficacy on the relationship between life‐to‐academic program conflict and academic performance

**FIGURE 2:**
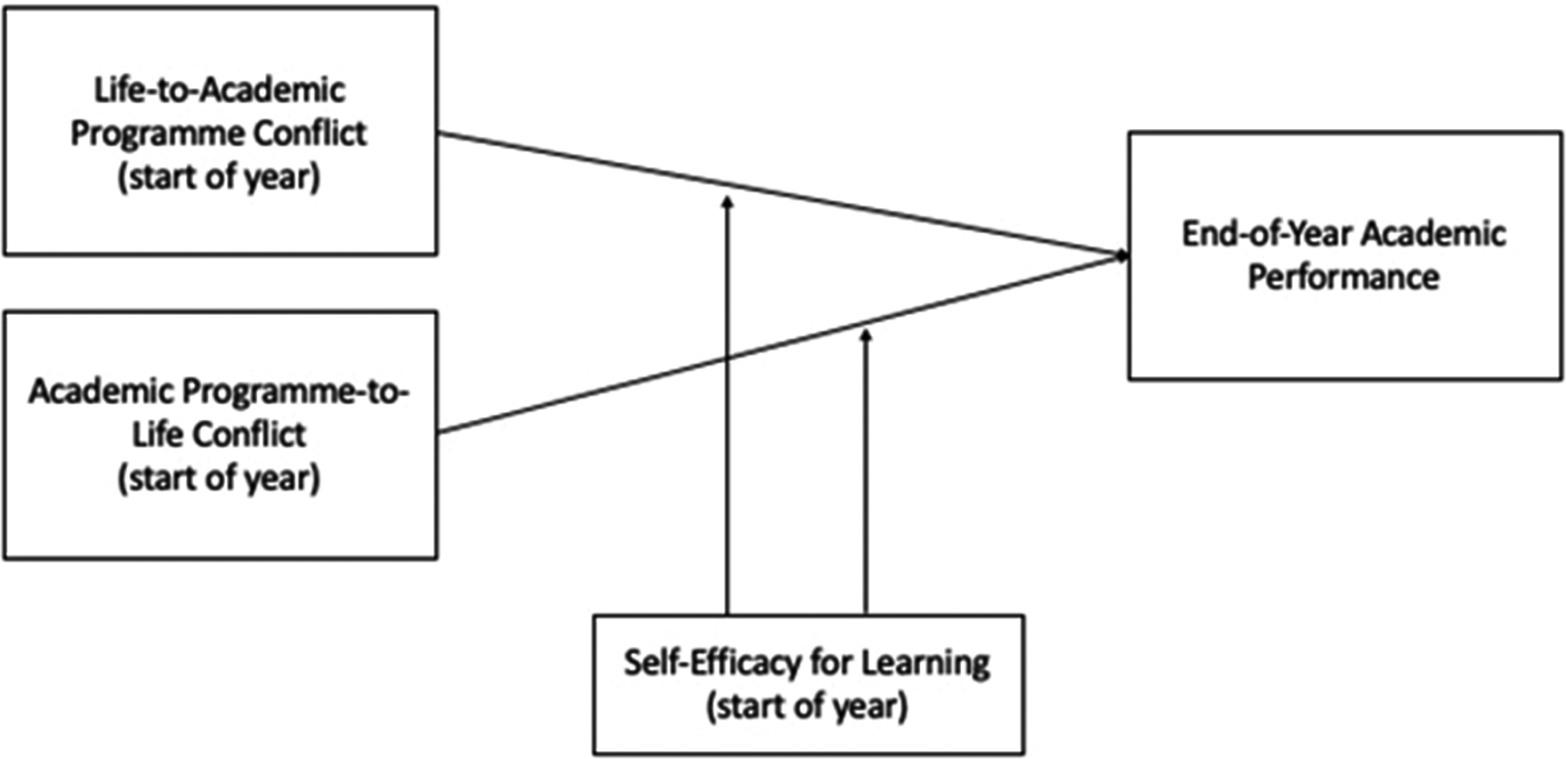
Research model

We investigated the buffering effect of self‐efficacy for learning by examining whether it moderated the relationships between both forms of role conflicts and academic performance. Self‐efficacy for learning significantly moderated the negative relationship between LAPC and end‐of‐year academic performance (see Table 3), while controlling for APLC, in support of Hypothesis 3(a), but we did not find this relationship for conflict originating from one's academic program towards one's life roles (see Table 4). As such, for the significant interaction, we ran a simple slopes test to analyze the conditional effects using low and high levels of the moderator (see Figure 1). Post hoc analysis revealed that conflict from one's life roles to one's academic program activities has a stronger relationship with academic performance when self‐efficacy is low, *b* = −0.14, *t*(61) = −2.98, *p* = 0.004, or moderate, *b* = −0.08, *t*(61) = −2.46,*p* = 0.017 (see Figure 2).

## 4 DISCUSSION

This study set out to investigate whether role conflict (i.e., conflict between life responsibilities and academic work) has a negative impact on a student's academic performance, and whether learning self‐efficacy buffers this negative impact. Our findings showed that LAPC was negatively associated with end‐of‐year academic performance, while APLC at the beginning of the year was not. We also found that one's self‐efficacy for learning buffered the negative effects of the conflict from one's life role to one's academic work on academic performance. Thus, graduate entry students with higher self‐efficacy for learning in the early stages of their studies were better able to mitigate the impact of such conflict so that their end‐of‐year academic performance was not impacted to the same extent as those with lower self‐efficacy for learning. Learning self‐efficacy is a domain‐specific construct which pertains to beliefs concerning one's capabilities to learn or perform behaviors at designated levels (Schunk, 1996). In this context, it could be considered advantageous for students to start their graduate entry program with high belief in their ability to learn, as it could potentially motivate them to persevere for longer and doubt themselves less (e.g., You, 2018).

Students in graduate entry programs are thought to have substantial life experience, which is desirable as this also means they likely bring maturity and interesting perspectives to their courses and subsequent roles (Gibbons, 2010, Rapport et al., 2009). At the same time, students entering these programs may also have increased home and life responsibilities. Limited research has been conducted to examine the relationship between role conflicts and student performance, although there is some evidence to suggest such relationships from past research focusing on adult learners and postgraduate students (Choo et al., 2021; McNall & Michel, 2011). The findings of our study demonstrate that the presence of higher home‐life demands at the start of the program has a negative impact on a student's overall academic performance. From a practical perspective, these findings emphasize the importance of accounting for and addressing added home‐life pressures of students entering graduate entry programs by putting structural supports in place. Drawing on previous inter‐role conflict literature, key predictors of perceived work–life balance are flexible work schedules and supportive workplace culture (Jang & Zippay, 2011). Thus, graduate entry programs should endeavor to accommodate flexible learning where possible. For example, they could provide online resources for those who cannot attend in person, or allow flexibility around submission deadlines.

In line with past research (e.g., Chemers et al., 2001; Dogan, 2015; Talsma et al., 2018), our findings demonstrated that self‐efficacy for learning was directly associated with academic performance. In addition, the results indicated that self‐efficacy buffered the negative relationship between LAPC and academic performance such that when student self‐efficacy was moderate or high, LAPC did not have as much of a negative effect on their academic performance, compared to low self‐efficacy for learning. These findings suggest that it would be beneficial for graduate entry programs to put in place strategies to build self‐efficacy early in the program. There are well‐established mechanisms to build self‐efficacy, which include mastery activities, vicarious learning, and verbal persuasion (Bandura, 1997; Schunk & Pajares, 2002), and would be likely to positively impact students’ academic performance (Bandura & Locke, 2003; Pajares, 1996). Van Dinther et al. (2011) conducted an empirical review which concluded that it is possible to increase student self‐efficacy within higher education programs. Of the different treatment modalities available, participant modeling has been found to be particularly efficacious (Feltz et al., 1979; Schunk, 1989). As such, one way to support student self‐efficacy might be to incorporate role modeling from past graduates who successfully navigated role conflicts during their graduate program.

Internationally, a number of health professional programs have attempted to address factors related to burnout, stress, and resilience in their curricula through the inclusion of educational workshops early in students' academic programs (Bird et al., 2020; Johnson et al., 2020; Kreitzer & Klatt, 2017; Wu & Oprescu, 2021). Some studies recommend a broader perspective, claiming that all aspects of teaching and learning need to be considered; for example, the manner in which educators provide feedback and communicate with students can help to improve self‐efficacy, and also peer support can be utilized within teaching and learning initiatives and induction material in order to facilitate coping strategies (Gibbons, 2010; O'Connor & McCurtin, 2021). While these educational supports and strategies have been reported as useful by students, it may also be worth considering the strategic timing of these supports, in order to maximize their impact. Our findings suggest that role conflicts are important to address very early in the graduate program. It would be useful to provide mentorship, support, and education at those times so that students can apply these strategies in a timely manner.

### 4.1 Limitations

Although our findings demonstrate that conflict from one's life to one's academic studies impacts academic performance across a wide range of graduate entry programs in medicine and allied health domains, the findings are limited in that respondents originated from one university. Further, the sample size is small, which may have been impacted by the Covid‐19 pandemic, as the even though study had been initiated in advance of Covid‐19, the second and third time points occurred during the early pandemic crisis when academic teaching moved to online learning. This transition may have caused students to focus more on negotiating these academic challenges which may have had an effect on participant recruitment at the second and third time points. Future research should be conducted to establish the generalizability of our findings to other samples and other countries.

There were more females than males in our sample, which is consistent with the gender ratio of the health professional student groups involved. Nonetheless, our findings must be interpreted in light of this. Most participants declared no caring responsibilities. However, our preliminary analysis revealed that those who did have caring responsibilities reported significantly higher levels of LAPC at the beginning of the academic year in comparison to those without caring responsibilities, which is as we would expect. Our measure of role conflict also incorporated other roles of responsibility, such as home and recreational roles and responsibilities, which would have also demanded time away from their studies. Given our aims (focusing on role conflict rather than specific forms of role conflict) and the nature of our measure, we cannot distinguish between specific manifestations of role conflict, although this may be an interesting angle for future research to explore in more detail.

## 5 CONCLUSION

This study indicates that higher levels of self‐efficacy are both directly and indirectly beneficial for student academic performance. Our findings suggest that high levels of self‐efficacy for learning can positively influence negative relationships between LAPC and end‐of‐year academic performance. Increasing student awareness in advance of initiating study on graduate entry programs would be helpful to ensure that students are aware of potential challenges, and have strategies to deal with them. Further, it may be beneficial to equip graduate entry students early in their program of study with skills designed to improve self‐efficacy. This may be achieved through a review of current teaching practices used across graduate entry programs, incorporating self‐efficacy treatment modalities, such as modeling experiences, to help strengthen student self‐efficacy. Additionally, graduate entry program providers should take into consideration the added life responsibilities of these student cohorts, as highlighted by our findings, and work towards facilitating more flexible learning opportunities where possible, thus making these programs more accessible.

## CONFLICT OF INTEREST

None declared.

## AUTHOR CONTRIBUTIONS

*Study design*: Anne O'Connor and Deirdre O'Shea. *Data collection*: Anne O'Connor. *Data analysis*: Deirdre O'Shea and Gemma McCarthy. *Manuscript writing*: Anne O'Connor, Gemma McCarthy, and Deirdre O'Shea.

## Data Availability

Data available on request from the authors
